# Spatiotemporal coordination of virulence, metabolism, and stress responses shapes infection dynamics of *Xanthomonas perforans*

**DOI:** 10.1128/aem.00681-26

**Published:** 2026-07-27

**Authors:** Amanpreet Kaur, Sivakumar Ramamoorthy, Palash Ghosh, Kylie Weis, Neha Potnis

**Affiliations:** 1 Department of Entomology and Plant Pathology, Auburn University 1383, Auburn, Alabama, USA; The University of Tennessee Knoxville, Knoxville, Tennessee, USA

**Keywords:** adaptation strategies, spatiotemporal coordination, pathogen transcriptional profiling, functional programming, *Xanthomonas perforans*, *in planta *transcriptome

## Abstract

Plants host diverse microbial communities across spatially distinct foliar niches. For hemibiotrophic pathogens, the epiphytic leaf surface and the apoplast impose contrasting physiological constraints that shape infection strategies. We investigated how these environments program the transcriptional responses of a tomato pathogen, *Xanthomonas perforans* (*Xp*), and how virulence, metabolism, and regulation are coordinated across space and time during disease progression. By selectively enriching and profiling the transcriptome of a pathogen from the leaf surface and apoplast, we uncovered pronounced niche-specific and colonization stage-specific gene expression patterns. Early epiphytic colonization features activation of chemosensing and motility to promote relocation, alongside iron and phosphate acquisition, DNA and protein repair, quorum sensing, phenylalanine degradation/tyrosine conversion consistent with countering phenylpropanoid defenses, osmotic and oxidative stress mitigation, DNA exchange machinery, and type VI secretion-mediated microbial competition. Upon entry into the apoplast, *Xp* shifts toward active metabolism and replication, accompanied by investment in type II and III secreted virulence factor expression, together with countermeasures against host-mediated nutrient sequestration. Late apoplastic infection triggers renewed stress responses and reactivation of motility, consistent with preparation for dissemination. Across both niches, oxidative and nutrient stress responses are prominent, but the specific gene sets engaged differed by environment. Together, this work provides a mechanistic, systems-level model of how a foliar pathogen perceives and adapts to structured host niches, revealing spatiotemporal regulatory logic that coordinates survival on the leaf surface with virulence and growth in the apoplast. This framework provides broadly applicable insights into ecology and virulence programming *in planta*.

**IMPORTANCE:**

Foliar bacterial pathogens face a series of shifting challenges as they move from the leaf surface into the protected interior. *Xanthomonas perforans* is a major tomato pathogen, yet how it engages in molecular dialog with the host has remained largely unclear. Reverse genetics approaches have revealed individual virulence factors, but they capture only fragments of a much larger process. By profiling pathogen transcriptomes in distinct microhabitats, we show how *X. perforans* reprograms its physiology during infection, adjusting motility, stress tolerance, metabolism, and virulence. This stage-resolved perspective uncovers the logic behind the infection cycle, revealing coordinated gene expression programs and regulatory modules during niche transitions. Our findings also demonstrate that ecologically similar foliar pathogens do not solve the same problems in the same way. Although *X. perforans* and *Pseudomonas syringae* occupy comparable niches, they adopt distinct metabolic and stress-response strategies, and plants perceive and respond to them differently. Such contrasts highlight how even closely related infection lifestyles can trigger unique molecular dialogs with the host. By illuminating nutrient limitations, and regulatory programs that shape infection from the outset, this work provides a systems-level view of disease establishment and identifies vulnerable points in the infection cycle that could be leveraged for targeted disease management.

## INTRODUCTION

Plants present multi-compartment niches for diverse microbes, including both beneficial and detrimental ones that influence growth and overall productivity. Each niche is shaped by distinct physical, chemical, and biological constraints (1–3). For foliar microbes, leaf surfaces and the extracellular space inside plant tissue (apoplastic space) represent two ecological niches. The leaf surface is vast, nutritionally poor, and environmentally variable with exposure to UV radiation, fluctuating humidity and temperature, episodic desiccation; and microbial competition for space and resources (4). On the other hand, the apoplast shields microbes from UV radiation and desiccation, offering opportunities to exploit host resources for multiplication while simultaneously challenging the pathogens with immune defenses and antimicrobial compounds (3). On leaf surfaces, bacteria contend with nutrient and water limited conditions, where even simple carbon sources and amino acids can be spatially patchy and diffusion limited (5). To locate these nutrient oases and withstand environmental stresses, bacteria rely on motility, chemotaxis, and biofilm formation (6–10). In addition, coordinated polysaccharide production, osmo-tolerance and stress response programs further enhance bacterial survival under these constraints (11–13). Epiphytic persistence is not merely passive but is a critical determinant to disease outcome, as surface population size is correlated with host entry and subsequent symptom development (14, 15). Entry into leaves typically occurs through natural openings such as stomata or hydathodes. Stomata actively participate in the innate immune response, which pathogen can overcome by deploying toxins and effectors to circumvent stomatal closure and gain access to the apoplast (16–18). Once inside the apoplast, the pathogen population expands, and disease is often associated with sites harboring large internal bacterial populations (19). Water availability becomes a central bottleneck in the apoplast. Successful pathogens manipulate host processes to transiently hydrate the apoplast (water-soaking), thereby facilitating bacterial proliferation. In *Pseudomonas syringae*, effectors, AvrE1 and HopM1 are known to induce apoplastic water-soaking (11, 20). In *Xanthomonas*, transcription-activator-like effector, AvrHah1, was shown to induce water-soaking and promote proliferation (21).

Because leaf surface and apoplast impose fundamentally different constraints, successful foliar pathogens must reprogram gene expression across space and time. In *P. syringae*, epiphytic stages emphasize active relocation in response to nutrients and water, whereas apoplastic growth favors metabolic reconfiguration, secretion system deployment, and stress tolerance (11, 15, 22). Early *in planta* transcriptomics further shows that plant immunity reshapes pathogen iron acquisition, secretion systems, and stress responses, linking bacterial gene expression states to later disease outcomes (23). Similar niche-dependent patterns were observed in *Xanthomonas campestris* pv. campestris during hydathode infection (24), including repression of motility and induction of sulfur and phosphate metabolism. These studies revealed that the driving forces for pathogen adaptation to epiphytic and apoplastic environments are distinct, emphasizing the importance of capturing the gene expression across both spatial and temporal stages.

**Fig 1:**
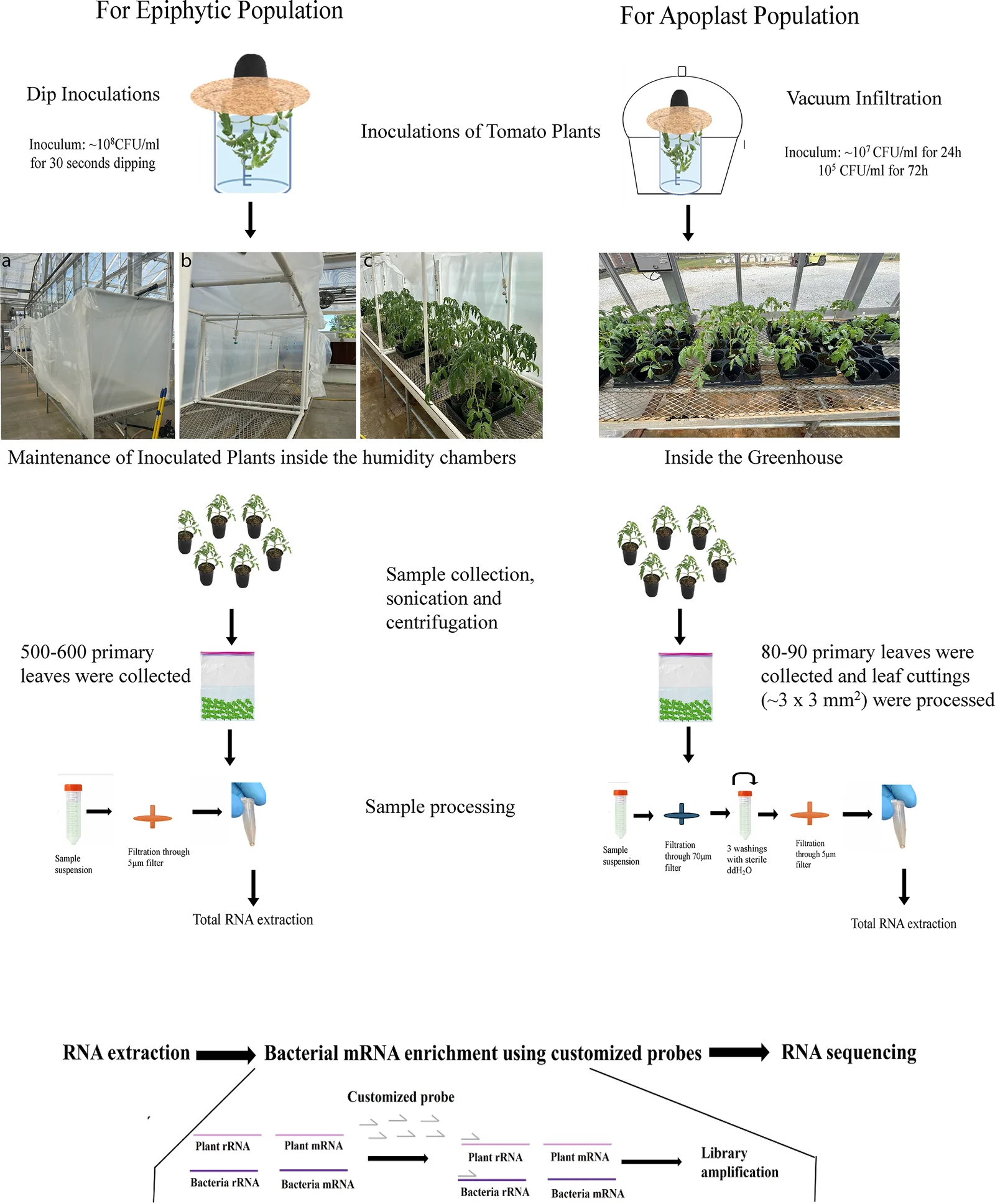
Experimental design used to establish bacterial populations in epiphytic and apoplastic niches. Epiphytic populations were established by dip inoculation with an inoculum concentration of ~10⁸ CFU/mL. Plants were maintained in humidity chambers equipped with an overhead sprinkler irrigation system (panels a–c), and sampled at 8 and 24 h post-inoculation. These chambers were built with PVC pipes and covered with transparent polyethylene sheets from all sides. In contrast, apoplastic populations were established by vacuum infiltration using initial concentrations of 10⁷ and 10⁵ CFU/mL for sampling at 24 and 72 h post-inoculation time points, respectively. Sampling was performed and followed by filtration to remove plant material. For epiphytic samples, a 5-µm filter was used, whereas apoplastic samples were first passed through a 70-µm filter and then a 5-µm filter to remove host tissue from leaf cuttings, followed by sonication. Total RNA was then extracted, and host-specific depletion probes targeting rRNA regions were used in the ribodepletion step. A list of custom probes is included in Table S1.

The genus *Xanthomonas* comprises a diverse group of Gram-negative bacteria causing disease in over 400 plant species and yield losses in major economically important crops (25). Although individual virulence factors, primarily, secretion systems (type I to type VI) and their associated effectors, along with exopolysaccharides and quorum-sensing systems, have been well characterized in *Xanthomonas* (25, 26), how their deployment is coordinated across space and time during infection is far less clear. *Xanthomonas perforans* (*Xp*) is also a hemibiotrophic seedborne pathogen within the genus *Xanthomonas*, and the causative agent of bacterial leaf spot (BLS) disease in tomato and pepper. *Xp* has a pronounced epiphytic phase, allowing the pathogen to establish on the leaf surface before entering the biotrophic phase (27). To elucidate the spatiotemporal adaptive strategies of *Xp* during tomato colonization, we conducted genome-wide transcriptome profiling of *Xp* strain AL65 across spatial niches of leaf surface (at 8 and 24 h after dip-inoculation) and apoplastic space (24 and 72 h after infiltration), with three biological replicates per time point (Fig. 1). For epiphytic populations, the 8 h time point was selected to capture the initial stages of adaptation to the leaf surface from *in vitro* culture, whereas the 24 h time point was chosen to represent a later stage of epiphytic colonization after exposure to phyllosphere-specific conditions. This spatiotemporal design enabled us to capture transcriptional reprogramming associated with early and late stages of infection. We leveraged weighted gene co-expression network analysis (WGCNA) to define niche- and time-specific transcriptional programs employed by *Xp* during tomato colonization, to probe the host environment perceived by the pathogen, and to understand how the pathogen modulates it during the infection process. We centered our interpretation on biologically informative genes within each co-expression module. Key genes were organized into functional categories based on KEGG pathway enrichment, and their differential expression was corroborated using differential expression analysis, enabling integrated interpretation of co-expression patterns and expression levels.

## RESULTS AND DISCUSSION

### *Xp* demonstrates niche-specific expression patterns during colonization and proliferation in epiphytic and apoplastic environments

**Fig 2:**
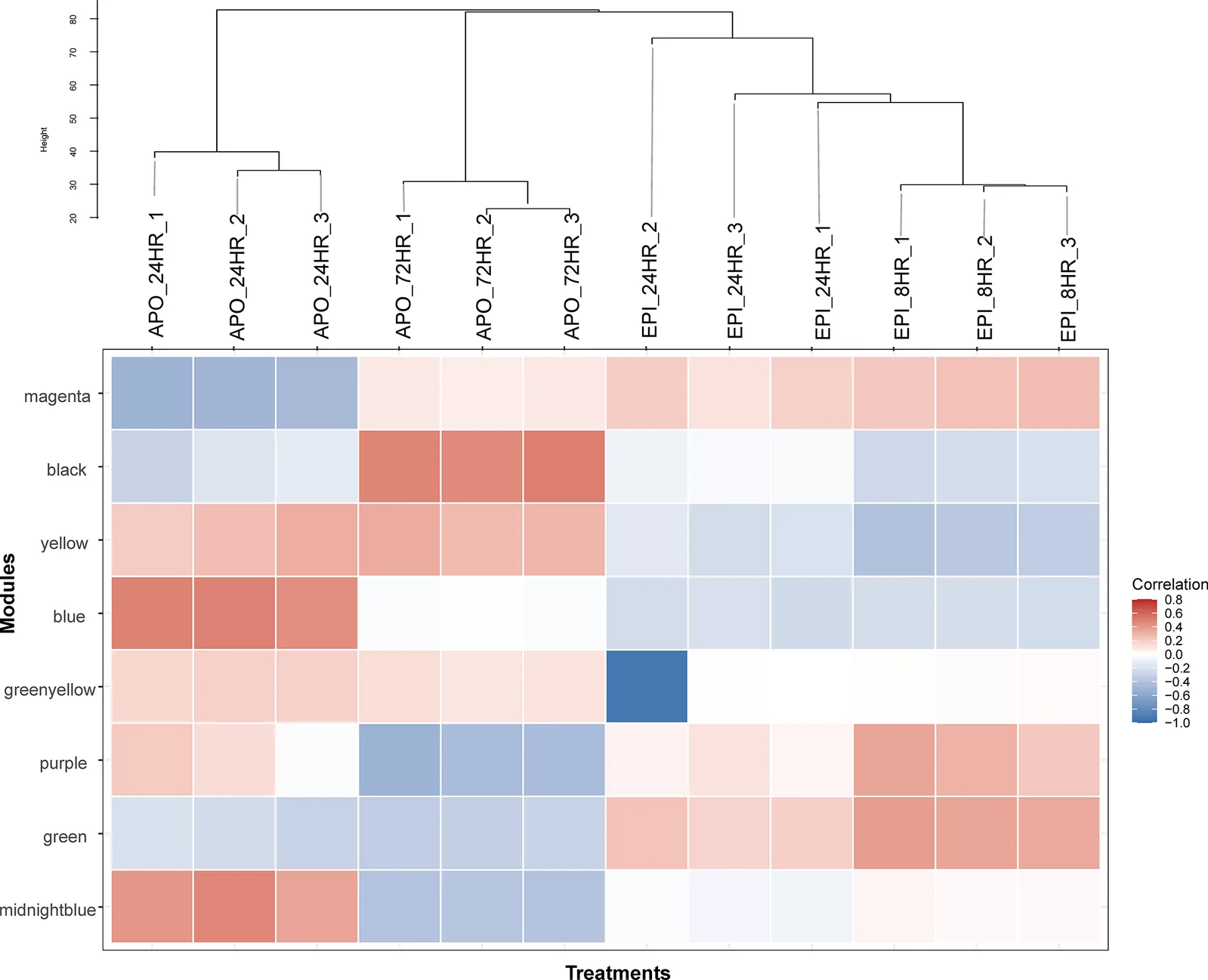
WGCNA analysis. The heatmap illustrates the modules identified by weighted gene co-expression network analysis (WGCNA), which groups genes based on pairwise expression similarities. The *y*-axis shows the different modules (blue, green, yellow, purple, magenta, midnight blue, green yellow, and black), while the *x*-axis represents the samples arranged according to hierarchical clustering. The color scale indicates the correlation between each sample and each module: darker red corresponds to higher correlation, implying stronger expression of the module genes in those samples, whereas lighter colors indicate weaker associations. The labels on the *x* axis (also on the dendrogram tip) refer to samples, where APO means apoplastic and EPI means epiphytic samples, with time points given and 1, 2, and 3 indicating replicate numbers for each specific treatment.

**Fig 3:**
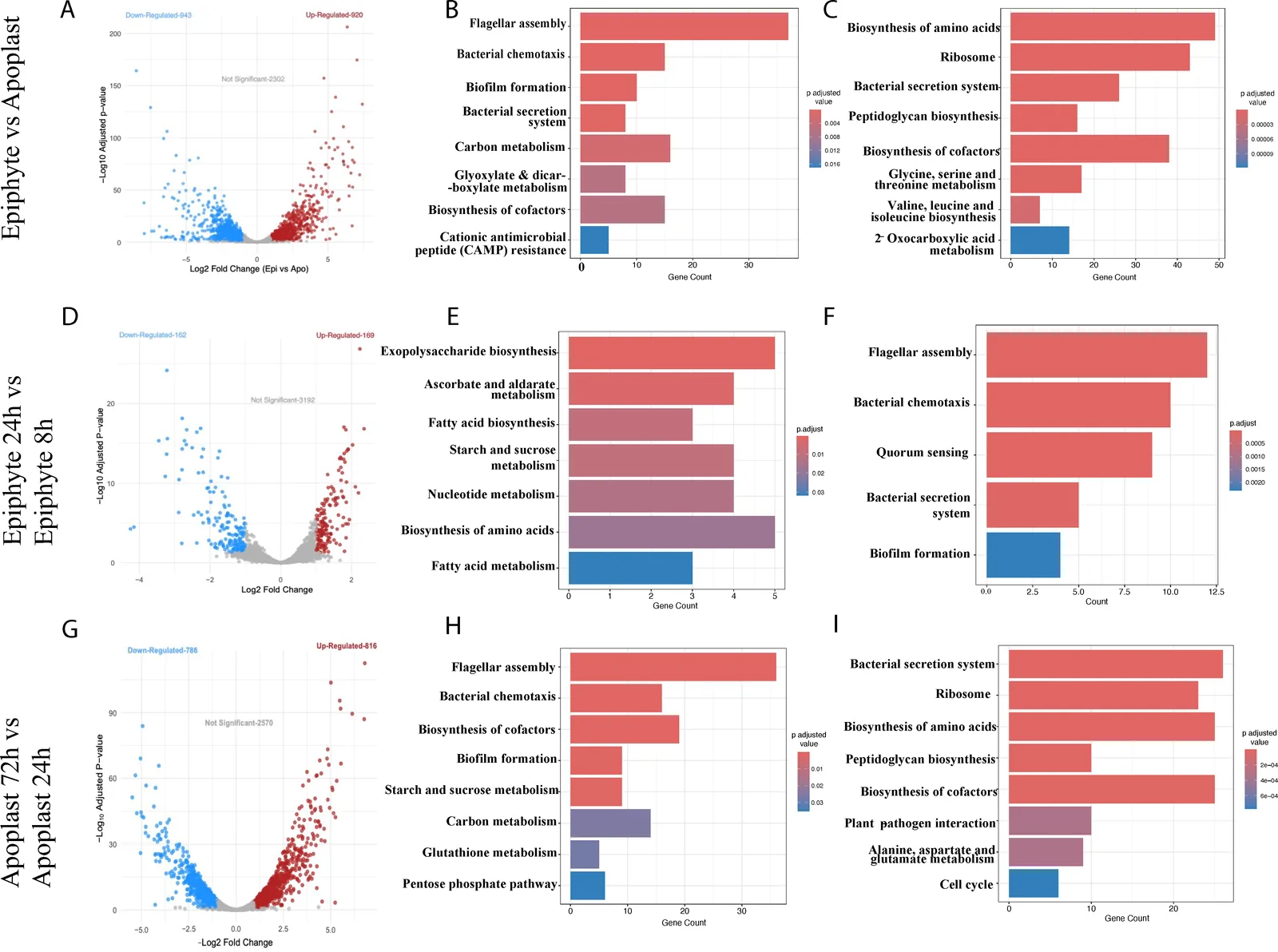
Transcriptomic analysis of *Xanthomonas perforans* (*Xp*) during colonization of epiphytic and apoplast niches in tomato plants. (**A, D, G**) Volcano plots showing differentially expressed genes (DEGs) of Xp under the following comparisons: epiphytic vs apoplast niches; epiphytic at 24 h vs epiphytic at 8 h; and apoplast at 72 h vs apoplast at 24 h, respectively. Up- and downregulated genes are indicated by red and blue dots, respectively. (**B, E, H**) KEGG pathway enrichment analysis of upregulated DEGs for the corresponding comparisons, with color indicating the adjusted *P*-value. (**C, F, I**) KEGG pathway enrichment analysis of downregulated DEGs for the same comparisons, with color indicating the adjusted *P*-value.

To identify coordinated gene expression patterns, we applied weighted gene co-expression network analysis (WGCNA), which clusters genes into modules based on highly correlated expression profiles. This approach revealed seven distinct modules (Fig. 2), each representing functionally related gene sets. Modules were prioritized for interpretation based on strong correlations with specific infection stages and niches. After filtering for high module membership (kME ≥ 0.8, *P*-value < 0.05), we identified 311 genes in the black module, 509 in blue, 514 in green, 141 in greenyellow, 341 in magenta, 120 in midnight blue, 136 in purple, and 289 in yellow. Notably, the green and yellow modules were niche-specific, corresponding to epiphytic and apoplastic conditions, respectively. Within the apoplast, the blue and midnight blue modules were associated with early infection (24 h), whereas the black module represented late infection (72 h). No temporal separation was evident during epiphytic colonization using WGCNA-associated modules; however, some differentially expressed genes were identified by DESeq2 analysis (Fig. 3). The purple module marked colonization of the epiphyte and early apoplastic niche, whereas the magenta module represented overlapping expression during late apoplastic and epiphytic colonization. Differential expression analysis using DESeq2 in parallel corroborated module-level trends observed in WGCNA. Using DESeq2, we observed that 920 genes were upregulated, and 943 genes were downregulated in epiphytic conditions compared with the apoplast. Further, 169 genes were upregulated and 162 were downregulated at 8 h compared with 24 h in the epiphytic niche, whereas 816 genes were upregulated and 786 were downregulated at 72 h compared with 24 h in the apoplast niche (Fig. 3). Interestingly, the cells recovered 24 h after dip-inoculation exhibited substantial transcriptional differences relative to those recovered 24 h after vacuum infiltration, with 2,017 genes differentially expressed (989 upregulated and 1,028 downregulated). Given that dip-inoculation primarily establishes bacteria on the leaf surface, populations at 24 h would remain largely epiphytic, although some may have entered substomatal chambers by 24 h (28). On the other hand, vacuum infiltration directly introduces bacteria into the apoplast. Thus, observed transcriptional divergence between 24 h after dip-inoculation and 24 h after vacuum infiltration is consistent with bacterial populations occupying distinct niches, epiphytic and apoplastic, with limited overlap between these conditions at this time point. To gain insight into the biological processes associated with these transcriptional changes, KEGG pathway enrichment analysis was performed on the differentially expressed genes (DEGs), and the significantly enriched pathways are summarized in Fig. 3 (Table S2).

Here, we focused on WGCNA modules to define niche- and time-specific transcriptional programs employed by *Xp* during tomato colonization, to probe the host environment perceived by the pathogen, and to understand how the pathogen modulates the host environment during the infection process. To further validate the association and direction of transcript expression, we examined the DESeq2 log2 fold-change (log2FC) values for the genes significantly associated with each module (Table S3). The key genes within each module were further functionally categorized, and their expression levels were confirmed using DESeq2 log2FC with values, which are listed in Table S4.

### Traits specific to the leaf-surface niche in *Xp* include chemotaxis, quorum sensing, motility, conjugal transfer, cellulose synthesis, active nutrient acquisition, DNA and protein repair, metabolism, and stress-tolerance mechanisms

The epiphytic niche was defined by the green module (514 genes), with 440 genes significantly upregulated relative to the apoplast (adjusted *P* < 0.05). Temporal differentiation was minimal, as only 157 genes were upregulated, and one gene was downregulated at 8 h compared with 24 h, indicating largely stable epiphytic expression across time points. The log_2_FC values reported here represent the expression changes under epiphytic conditions compared with apoplastic conditions, unless otherwise specified, when comparing time points (also see Table S4 for log_2_FC).

#### Epiphytic colonization involves a coordinated transition from environmental exploration to surface attachment

During early epiphytic colonization (8 h), a strong induction of chemotaxis genes indicates active environmental sensing. Of the 48 methyl-accepting chemotaxis proteins (MCPs) encoded in the genome, 22 were assigned to the epiphytic-specific green module and were strongly upregulated relative to the apoplast (ranging between log_2_FC: 1-5). MCPs showing the highest induction (log_2_FC: 4–5) were frequently adjacent to genes encoding hypothetical proteins. Multiple flagellar genes, including *motB2* (E2P69_RS04570), *motA* (E2P69_RS04575), and *fliJ* (E2P69_RS17055), were represented in the green module and were upregulated under epiphytic conditions (log_2_FC: 3–4). MotA and MotB form the stator complex driving flagellar rotation, FliJ contributes to flagellar assembly and virulence; however, average levels of these flagellar transcripts declined at 24 h of epiphytic growth relative to 8 h (avg. log_2_FC: –1.1), indicating a progressive shift away from active swimming as surface colonization proceeds (29, 30). Notably, two MCPs, E2P69_RS20880 (log_2_FC: 2.911) and E2P69_RS20895 (log_2_FC: 3.708), flank the flagellar brake gene, *ycgR* (log_2_FC: 3.156). YcgR functions as a cyclic di-GMP (c-di-GMP)-responsive “backstop brake” that modulates flagellar motor direction and speed, enabling fine-tuning of swimming velocity in response to environmental cues (31, 32). This genomic organization suggests MCP-guided regulation of flagellar motility under epiphytic growth. Genes involved in type IV pili biogenesis (*pilGHIJTUMNAWT*) were strongly upregulated in the epiphyte niche compared with the apoplast. These genes were co-expressed with the twitching motility response regulators *pilG* and *pilH (*which negatively regulate swimming motility and positively regulate swarming motility, log_2_FC: 1.20 and 0.99, respectively, relative to 24 h), indicating coordinated regulation of surface-associated and collective motility during early epiphytic colonization (33). While most chemotaxis and chemosensing genes were significantly more highly expressed at 8 h than at 24 h, *pilJ* and *pilI* showed no temporal differences, suggesting sustained mechanosensory signaling.

Upregulation of cellulose synthase (*bcsABC,* log_2_FC: 2.159) was observed in this module. The operon *bcsABC,* a core component of the cellulose biosynthesis systems of most Gram-negative bacteria, is activated by c-di-GMP to bind to the PilZ domain, thereby promoting biofilm production (34). Quorum sensing and c-di-GMP signaling were also prominently regulated under epiphytic conditions. The entire *rpf* operon clustered in the green module was significantly upregulated relative to the apoplast, indicating a coordinated regulatory response. The *rpfF* gene was upregulated at 8 h (log_2_FC: 0.857 relative to 24 h), suggesting early DSF production, whereas *rpfB, rpfC,* and *rpfG* genes were consistently upregulated (log_2_FC: 0.82, 0.922, and 0.798, respectively), with no significant temporal differences. Early rising levels of c-di-GMP followed by induction of *rpfG* suggest a transition from motile to sessile state, and modulating biofilm formation. Co-expression of three GGDEF-domain diguanylate cyclases, two EAL-domain phosphodiesterases, and one HD-GYP-domain protein indicates fine-scale modulation of c-di-GMP homeostasis. An additional regulator linked to c-di-GMP signaling, the transcriptional activator *zur*, was significantly upregulated under epiphytic conditions (log_2_FC: 2.0395) (35).

Thus, although flagellar genes are transcriptionally upregulated to enable initial exploration, our data showing suppression of flagellar gene expression by 24 h support a coordinated regulatory strategy, in which concurrent c-di-GMP signaling drives simultaneous induction of *ycgR* (flagellar brake) and cellulose biosynthesis. Thus, even when flagellar genes are expressed, their activity is likely constrained, consistent with the role of YcgR as a c-di-GMP-responsive “molecular wheel locks” that inhibits flagellar rotation (36, 37), while cellulose biosynthesis can further impose physical constraints on motility and promote surface attachment (38). This regulatory configuration supports a model in which motility is finely tuned from free-swimming to surface-associated movement, mainly relying on type IV-pili mediated twitching, enabling short-range exploration and microcolony formation on heterogeneous leaf surfaces, especially when conditions on the leaf surface are dry, and the bacterium may not be able to use flagella, and most of the stomata are closed (39). Type IV-pili-mediated aggregation may improve bacterial fitness on the leaf surface (40, 41). Simultaneously, cellulose production stabilizes attachment, enhances stress tolerance, and seeds early biofilm formation.

#### Leaf surface colonization promotes microbial interactions and genetic exchange

Genes encoding the type IV secretion system (T4SS) were strongly expressed during early epiphytic colonization. WGCNA placed *virB1, virB7–virB10*, *virC1,* and *virD4* in the epiphytic-specific green module, while *virB1, virB11,* and *virB4* clustered in the purple module associated with epiphyte infection and early apoplast niches. Plasmid-borne genes involved in conjugation, *trbD* and *trbB*, were significantly upregulated at 8h epiphytic growth (Table S1), and additional transfer genes (*trbCEFGIJKL*) were associated with epiphytic and early apoplastic networks (purple and midnightblue modules). The transcriptional regulator *traJ* was also upregulated (log_2_FC: 1.4), suggesting that horizontal gene transfer is favored during leaf-surface colonization. Genes involved in natural transformation including genes encoding competence proteins, ComEA, ComF, DprA (SMF, a DNA-processing protein), genes encoding SMG proteins involved in nonsense-mediated RNA decay (post-transcriptional regulation), were co-expressed (42, 43). A gene encoding HU DNA-binding protein that facilitates biofilm formation by anchoring extracellular DNA (eDNA) to bacterial cells was co-expressed, indicating a potential role in stabilizing biofilm architecture and promoting genetic exchange on the leaf surface (44).

#### Epiphytic cells experience extensive environmental and host-derived stresses

Epiphytic cells expressed a broad stressome encompassing oxidative, osmotic, and envelope stress responses, along with multidrug efflux (*emrA*, *emrB*, up to 3.7-fold in epiphyte relative to apoplast, and log_2_FC ~ 1 at 8 h compared with 24 h) and an AlgE-family outer membrane protein (putative exopolysaccharide export).

##### Oxidative stress responses

Key regulators included *ohr* and *ohrR* (organic hydroperoxide resistance), and a MarR family transcriptional regulator, involved in detoxifying oxidative byproducts. Catalase *katB,* involved in protecting cells from hydrogen peroxide toxicity, was induced (log_2_FC: 2.99). Unlike *Pseudomonas syringae* (22), which preferentially induces oxidative defenses in the apoplast, genes encoding oxidative stress enzymes (*ahpF, ahpC, ohr, katB,* and *sodA*) were more strongly induced on the leaf surface in *Xp.* This contrast highlights a fundamental difference in strategy: whereas *P. syringae* mounts a reactive oxidative stress response primarily after entering the apoplast, *Xp* activates this response early, suggesting either ROS pathway induction during initial colonization or anticipatory defenses on the leaf surface to withstand oxidative bursts before host entry. The OxyR regulon, comprising *oxyR*, *ahpCF*, *katB*, and *trxB* (45), was co-expressed, with higher transcript levels in epiphytic vs. apoplastic growth (log_2_FC ~ 2-4), indicating activation of hydrogen peroxide–inducible defenses. OxyR additionally regulates peroxiredoxin and thioredoxin reductase and has been implicated in T6SS regulation (46). The *cyoABCD* operon, encoding cytochrome-o-ubiquinol oxidase involved in redox balance and oxidative stress adaptation, was upregulated in epiphytic conditions. Deletion of these genes in *X. oryzae* increases sensitivity to hydrogen peroxide and reduces virulence (47). Additional redox genes included *yneN* (thioredoxin) and *rbn* (UPF0761 YihY family inner membrane protein), and *gshB* (glutathione synthase), which support glutathione-based detoxification. Green module also included gene encoding rubredoxin, an iron-sulfur protein involved in electron transfer, reduction of superoxide, and metabolism.

##### Osmotic stress response

Epiphytic cells upregulated *treA* (trehalase, enabling trehalose utilization under high osmolarity), and the high-affinity ATP-driven K^+^ uptake system (*kdpABCD*), forming an ATP-dependent K^+^ pump that maintains intracellular potassium levels and intracellular turgor pressure under osmotic stress (48, 49). Interestingly, this system was highly upregulated under epiphytic conditions, irrespective of time points, with transcript levels of about 4. Regulators, such as *ompR* (response regulator, activated upon sensing of changes in osmolarity by membrane-bound sensor kinase, EnvZ) and *osmC* (osmotic and peroxide stress protection), were also significantly upregulated with log_2_FCs of 2.49 and 1.8, respectively, compared with apoplast, indicating porin remodeling and peroxide/osmotic protection (50, 51).

##### Envelope stress response and intermicrobial competition

Copper homeostasis genes (*copA*, *copB*, and copper chaperone) and *baeS* (envelope stress sensor) were expressed, alongside mercury resistance. Interestingly, *baeS* is indicated to be activated in response to cell envelope damage, such as an attack from a competitor’s T6SS (52). T6SS components were distributed across modules (*tssM* in magenta; *hcp* in green, and *vgrG*/*vipA* in the green yellow module), with *tssM* significantly higher in epiphytic conditions (log_2_FC: 1.23), consistent with competitive interactions on the leaf surface. Some sigma-E regulon-related genes (*rpoE*, *rseA*, *mucD*), mediating adaptation to membrane-perturbing conditions (53), were unique to the epiphytic niche.

##### Protein folding/misfolding and DNA repair

Green module included genes involved in multiple DNA repair systems *radA* and *msrA* (peptide-methionine S-oxide reductase, which repairs oxidatively inactivated proteins), DNA repair photolyase, MiaB family radical SAM proteins, *fpg* (a bifunctional DNA-formamidopyrimidine glycosylase/DNA-(apurinic or apyrimidinic site) lyase), and the *msrPQ* system for repairing oxidized periplasmic proteins. Additionally, chaperones (*dnaK*, *grpE*, *hslO*) and proteases (*lon*, *degQ*) were co-expressed to prevent protein misfolding. Clp, an indirect regulator of GroEL/GroES, was also upregulated, along with phosphatidate cytidylyltransferase, which modifies membrane fatty acid composition under stress. DnaK and GrpE proteins prevent aggregation of stress-denatured proteins. DegQ family serine endoprotease, *mucD*, degrades transiently denatured and unfolded proteins, which accumulate in the periplasm following stress conditions. Genes involved in protein quality control and redox regulation, such as *hslO* and thiol-disulfide interchange proteins (*dsbA/L*), were expressed, ensuring proper folding of periplasmic and virulence proteins. The *msrPQ* system, repairs oxidized periplasmic proteins containing methionine sulfoxide residues, protecting against oxidative stress, while *lon* endopeptidase selectively degrades mutant, or abnormal proteins. Other co-expressed genes include *hslO* chaperone that is redox regulated and protects thermally unfolded and oxidatively damaged proteins. STRING network analysis linked *ahp* and *yneN* to these stress-response pathways (54). Genes encoding DNA recombination protein RmuC (anti-phage/anti-MGE), recombination regulator RecX, recombinase RecA, integration host factor alpha IhfA, were co-expressed in the green epiphytic module (typical log_2_FC ~1.2, *ihfA/recA* 0.8 at 8 h), consistent with IHF-mediated nucleoid remodeling that directly regulates promoters of type I-VI secretion system genes, as demonstrated in *Dickeya* (55). Co-expression of *dsbA/L* suggests DsbA/L-dependent disulfide bond formation, supporting folding of periplasmic virulence proteins and type III effectors during epiphytic colonization (56). NUDIX hydrolases, which contain an Hrp box and are therefore predicted HrpX targets, were expressed on the leaf surface together with NAD salvage genes, encoding nicotinamide-nucleotide adenylyltransferase and nicotinate phosphoribosyltransferase (log_2_FCs: 4.0815 and 3.2490, respectively), despite the absence of *hrpX* expression under epiphytic conditions. Stressosome-associated components, including *rsbR*, a STAS-domain protein, were also expressed, indicating activation of global stress signaling pathways during epiphytic colonization (57).

### The tomato leaf surface is nutrient-limited and chemically challenging, driving nutrient scavenging, metabolic adaptation, and detoxification responses

Transcriptional responses during epiphytic colonization indicate that *Xp* experiences substantial nutrient limitation while simultaneously encountering plant-derived antimicrobial compounds.

#### Nutrient acquisition and metabolic adaptation

Genes encoding at least nine TonB-dependent receptors (TBDRs), three energy transducers, and *exbD4* transporter were co-expressed under epiphytic conditions, indicating active scavenging of scarce nutrients. Early epiphytic upregulation of the gene encoding ferric-rhodotorulic acid/ferric-coprogen receptor *fhuE,* and phosphate-selective porins (*oprO/oprP*) suggests iron and phosphate limitation on the leaf surface (58, 59). Consistent with this interpretation, genes involved in iron homeostasis and FE-S cluster biogenesis, including *sufT, fdxA, i*scA*, hesB*, and *grxD*, were co-expressed throughout epiphytic colonization, supporting persistent iron-depleted conditions throughout epiphytic colonization (60–62).

To cope with the resource limitations, *Xp* exhibits broad metabolic programming. Metabolic pathways enriched epiphytically included aspartate 1-decarboxylase, which supports β-alanine production for pantothenate and CoA biosynthesis; phospho-2-dehydro-3-deoxyheptonate aldolase, which initiates the shikimate pathway for aromatic amino acid synthesis and phenylalanine degradation. Induction of *phhA* (log_2_FC: 2.3) and *hppD* (log_2_FC: 1.04) suggests active utilization of phenylalanine, a precursor in plant phenylpropanoid defense, converting it to tyrosine during colonization, and hints at phenylalanine degradation by *Xp* early during epiphytic colonization and before infection of the apoplast, similar to that observed in *P. syringae* (22, 63). Similarly, co-expression of *fadL* and *fadB*, involved in long-chain fatty acid uptake and β-oxidation, and the lipid-binding protein, DegV*,* suggests coordinated regulation of fatty acid storage and catabolism, potentially restricting fatty acid export via membrane vesicles and linking lipid metabolism to vesicle regulation in the epiphytic niche (64).

#### Exploitation of plant-derived substrates

In addition to scavenging nutrients, epiphytic cells expressed several cell wall-degrading enzymes (CWDEs), including glycoside hydrolases (GH5, cellulase A; GH30; GH39/*xynB*), and rhamnogalacturonan lyase. Early epiphytic growth (8 h) showed strong induction of a cellulase family protein (*E2P69_RS19010*), and two GH5 genes encoding endo-β−1,4-glucanases involved in cellulose breakdown (*E2P69_RS00220* and *E2P69_RS00235*), indicating active degradation of plant cell wall xylans (65).

#### Adaptation to plant defense metabolites

Plants deploy antimicrobial metabolites during pathogen attack, including volatile esters and aldehydes. During epiphytic colonization, *Xp* co-expressed *prpR* encoding propionate catabolic regulator, suggesting active metabolism of plant-derived propionate during epiphytic colonization, potentially counteracting stomatal closure and maintaining access to entry points (66). In addition to esters, plants emit volatile organic compounds such as methanol, which can form formaldehyde—a toxic intermediate that must be detoxified. Concurrently, formaldehyde detoxification genes (*frmR*, *frmA*, *gfa*, and *fghA*), including *gfa*, encoding 5-hydroxymethyl glutathione synthase, and an aryl aldehyde reductase (E2P69_RS15385), were strongly induced during epiphytic conditions, consistent with active detoxification of aldehydes and a robust glutathione-dependent system for mitigating aldehyde and oxidative stress on the leaf surface (67, 68). Eight genes encoding type II toxin–antitoxin (TA) system components were significantly overexpressed during epiphytic (avg. log_2_FC: ~1.5) growth relative to apoplastic conditions, with four induced specifically during early epiphytic colonization and remaining four throughout the epiphytic phase. TA systems are well established stress response modules that promote bacterial persistence (persister state) under nutrient limitation and oxidative stress by inducing reversible growth arrest or stabilizing essential pathways (69–71). These findings highlight how *Xp* integrates metabolic flexibility, detoxification mechanisms, and stress-adaptive strategies to thrive in the chemically challenging epiphytic niche.

### Initial contact and invasion of host cells prime horizontal gene transfer, and effector deployment

The purple module was strongly associated with pathogen adaptation from arrival on the leaf surface to transition to the apoplast niche upon host entry. Of its 136 genes, 126 showed no significant differences between epiphyte and apoplast at 24 h. Further, 44 were significantly upregulated during early epiphytic colonization (8 h vs 24 h), whereas 124 were induced during early apoplastic infection (24 h vs 72 h) (see also Table S4 for log_2_FC). This module included plasmid-borne genes involved in surface attachment, horizontal gene transfer, metabolism, and virulence, notably type IVa pilin genes (*pilO, pilP, pilQ*), T4SS components (*virB1, virB11*), conjugation genes (*trbE, trbI, trbJ* and recombinase), plasmid-borne TAL effector, *avrHah1* and chromosomal effectors (*xopQ* and *xopE),* all highly co-expressed at throughout epiphyte conditions and early apoplastic phase (mentioned above in epiphyte sub-section), with exception of conjugation genes (*trbC, trbF, trbG, trbL*) and other plasmid-borne effector *xopH* are expressed high under early colonization independent of niche. While *avrHah1* expression was significantly induced during epiphyte and early apoplast growth (log_2_FC: 2.32, compared with 72 h), other core effector transcripts were predominantly upregulated only during early apoplast infection. Central to this early transcriptional program was the global post-transcriptional regulator, *csrA,* which controls many physiological processes and virulence-associated traits (72–74) and is present in both chromosomal and plasmid-borne copies in AL65. Both copies were induced during early epiphytic growth, whereas only the plasmid-encoded *csrA* was upregulated during early apoplastic colonization, underscoring its likely role in coordinating niche-specific regulation of virulence, metabolism, and stress adaptation (25, 75). Additional co-expressed genes supported this early adaptive state in apoplast, including those involved in amino acid and nucleotide metabolism (*ggt, trpE, glyA, aroG*) and stress resistance (peroxiredoxin, alcohol dehydrogenase, and multidrug MFS transporters). This module also contained *acnB* (aconitase B), a [Fe–S] enzyme involved in both the conversion of citrate to isocitrate in the TCA cycle flux and redox/iron sensing (76, 77). These observations are consistent with the view that aconitases integrate metabolic demands with redox and iron homeostasis (78, 79). Given the abundance of organic acids, such as citrate in the tomato apoplast (80, 81), induction of citrate transporter (*citM*) (log_2_FC = 5.03) only in the early apoplast is consistent with a metabolic program that leverages apoplastic citrate during early colonization as previously reported (82, 83). Thus, early induction of *acnB* likely integrates metabolic readiness with stress sensing during initial host invasion.

### Apoplastic colonization involves a coordinated transition from environmental sensing to nutrient acquisition, virulence deployment, and adaptation to host-imposed stresses

In the apoplastic niche, the yellow and greenyellow modules were niche-specific, together comprising 430 genes, of which 299 were significantly upregulated relative to epiphytic samples. In contrast to the temporal stability observed during epiphytic colonization, apoplastic infection was strongly structured over time. Early apoplastic infection was dominated by the blue and midnight blue modules, in which 575 of 629 genes were significantly upregulated at 24 h compared with 72 h. Conversely, the black module was specific to late apoplastic infection, containing 311 genes, of which 308 were significantly upregulated at 72 h compared with 24 h (see also Table S4 for log_2_FC). Early infection is dominated by environmental sensing, nutrient acquisition, rapid growth, and virulence deployment, whereas later stages increasingly reflect adaptation to prolonged nutrient limitation, oxidative stress, and changing physicochemical conditions within infected tissues.

#### Environmental sensing promotes the transition to an endophytic lifestyle

Upon entering the apoplast, *Xp* rapidly activates sensory and regulatory networks that detect host-derived signals and coordinate the transition from surface-associated colonization to a virulence-associated lifestyle. Early apoplastic infection (blue and midnight blue modules) was enriched in genes involved in chemotaxis and signal transduction, including *raxH* and *cheW*. RaxH, a HAMP-domain histidine kinase, forms the RaxH/RaxR two-component system, which senses host-derived signals and regulates virulence-associated pathways. In *Xanthomonas oryzae*, this system controls *raxABC* type I secretion system, critical for virulence (84). Early apoplastic colonization was also marked by the induction of *guaA*, involved in nucleotide biosynthesis, and two EAL-domain phosphodiesterases (E2P69_RS08940 and E2P69_RS13670), suggesting active modulation of c-di-GMP levels. Consistent with this, c-di-GMP-responsive regulator Clp was active during early apoplast infection but was significantly downregulated by 72h. Because reduced c-di-GMP levels promote Clp activity and virulence gene expression (85). These observations suggest that apoplastic colonization is accompanied by a shift away toward a virulence-focused program. Additional apoplast-specific sensory systems included PAS-domain histidine kinases (E2P69_RS07440 and E2P69_RS07460) and the HAMP-domain kinase *colS*, orthologous to the *Pseudomonas* ColS/ColR system involved in metal sensing and low pH adaptation (86–88). The apoplast-associated yellow module contained *ravR*, an EAL-domain phosphodiesterase within the RavS/RavR two-component system, that promotes the transition from motility to virulence through c-di-GMP hydrolysis (89).

#### Environmental sensing remains important throughout disease progression

During later stages of apoplastic infection, expression of additional sensory kinases, including GAF-domain histidine kinase (E2P69_RS12270), two HAMP domain-containing histidine kinases (E2P69_RS01145 and E2P69_RS13405) and two PAS domain-containing histidine kinases (E2P69_RS04400 and E2P69_RS04625), suggests continued sensing and monitoring of changing host conditions as infection progresses. Overall, enrichment of EAL-domain enzymes in apoplast-specific modules indicates sustained c-di-GMP degradation during endophytic growth, whereas GGDEF-domain proteins associated with epiphytic modules suggest that c-di-GMP synthesis predominates during leaf-surface colonization.

#### The apoplast imposes nutrient limitation that drives extensive metabolic reprograming

Transcriptional landscape of apoplastic *Xp* indicates that entry into host tissues is accompanied by strong nutrient limitation, likely reflecting both the inherently restrictive nature of the apoplast and host-mediated nutrient sequestration as part of plant defense. In response, *Xp* activates coordinated programs for phosphate, sulfur, nitrogen, carbohydrate acquisition while simultaneously remodeling central metabolism to support growth, virulence, and stress adaptation. Phosphate-starvation responses were evident as early as 24 h, with induction of phosphate selective outer membrane porins (*oprO/oprP*, E2P69_RS01730/E2P69_RS15350), mediating uptake of orthophosphate and polyphosphate under starvation (59, 90, 91). High-affinity phosphate import was further reflected by co-expression of the *pstSCAB* ABC transporters dedicated to high-affinity inorganic phosphate (P_i_) uptake (92, 93) in both early and apoplast-specific modules, together with *pitA* (low-affinity P_i_ importer) and ppx (exopolyphosphatase that degrades polyphosphate to orthophosphate), indicating remodeling of P_i_ uptake and intracellular polyphosphate turnover in response to limitation (94). By 72 h, late apoplast samples showed co-expression of the phosphate-starvation regulators *phoR/phoB* and polyphosphate kinase (*ppk*), consistent with a shift from immediate uptake to regulatory and storage-based strategies characteristic of prolonged phosphate stress (95). Recent work emphasizes how cytoplasmic phosphate levels and PstSCAB flux jointly tune Pho activation, preventing inappropriate responses while enabling adaptation to P_i_ deprivation (92, 96). Activation of the Pho regulon at this stage suggests that *Xp* manages its fitness costs when P_i_ is locally sufficient (93) and activates it later during disease progression while adapting to host-mediated phosphate sequestration. A similar pattern was observed for sulfur metabolism. Pathogens rely on sulfur from their plant hosts and can manipulate plant sulfur transport systems to acquire sulfur during infection, while plants interpret sulfur depletion as a signal of pathogen invasion and intensify sulfur sequestration as a defense mechanism. This nutrient tug-of-war is critical because sulfur is essential for antioxidant defenses and redox homeostasis. While plants primarily assimilate inorganic sulfur as sulfate, bacteria can utilize both inorganic sulfate and organic sulfur such as sulfonates (secondary source under limiting conditions) and sulfate esters (97). In our data set, sulfur metabolism genes are largely enriched in the apoplastic niche, with the exception of a single SulP family inorganic anion transporter for sulfate uptake expressed epiphytically, contrasting with the predominantly epiphytic sulfur-uptake profile reported for *P. syringae* (22). Apoplast-specific modules revealed coordinated sulfur acquisition and assimilation programs. The yellow module contained *cysA* (sulfate/molybdate ABC transporter), while the early apoplast blue module included sulfate ABC transporter permeases (*cysW* and *cysT, log_2_FC:* ~0.94), sulfite reductase (*cysJIH,* log_2_FC: ~4.38), sulfonate ABC transporters (log_2_FC: ~3.77), and taurine catabolism genes (*tauA* and *tauD;* log_2_FC: 4.15 and 4.24) (reported log_2_FCs are relative to 72 h). The midnight blue module featured the sulfate-starvation regulator *cysB*, and sulfate adenylyltransferase (*cysC* and *cysD*), all showing strong induction (log_2_FC: ~3.12) at 24 h relative to 72 h. Because *cysB* activates sulfate starvation responses, including *tau* operon, this co-expression pattern suggests early sensing of sulfur limitation and a capacity to switch from sulfate to organic sulfur sources as stress intensifies (98). Similar induction of sulfonate and taurine utilization genes during PAMP-triggered immunity has been reported previously, consistent with host-mediated sulfur sequestration or oxidative damage to Fe-S cofactors, producing sulfur-starvation-like responses (99). Nitrogen acquisition pathways were likewise strongly induced during early apoplastic colonization (>2 log_2_FC higher expression relative to epiphytic and late apoplastic conditions). These included nitrate assimilation genes (*nasD, nasE*), nitrate reductase (E2P69_RS17555). Additional components, including an ammonium transporter (*amtB*), the P-II family nitrogen regulator (*glnB*), and glutamate synthase (*gltD*), were also upregulated at 24 h post-inoculation. This coordinated induction indicates active nitrate assimilation upon entry into the apoplast, likely reflecting nitrogen limitation imposed by host defenses. Similar niche-specific nitrate utilization has been reported in *P. syringae,* where nitrate reductase expression was observed exclusively in cells grown in apoplastic wash fluid, but not in macerated tissue, indicating nitrate utilization is a niche-specific adaptation (100). The presence of localized nitrate pockets on leaf surfaces was demonstrated using bioreporter experiments, with carbon remaining the primary limiting factor, followed by nitrogen (101). These observations suggest that a coordinated strategy for nitrogen assimilation and incorporation into amino acid biosynthesis during early infection, as host defenses often restrict nitrogen availability as part of immune responses.

#### Metabolic remodeling supports growth and virulence

Amino acid metabolism and translational capacity were actively remodeled during early apoplastic colonization. The blue module showed induction of phenylalanine-tRNA ligase subunits (*pheS/pheT*), consistent with a surge in translational demand to support rapid growth and the synthesis of virulence and stress-response proteins. Despite reported glutamate abundance in the tomato apoplast (102), *gltB* (glutamate synthase) was induced in the midnight-blue module, suggesting roles in ammonia assimilation, maintenance of central carbon-nitrogen balance, and redox control. Induction of *gltP* (glutamate/aspartate symporter) and glutamate-5-kinase further links glutamate metabolism to chemotaxis and osmoprotection via proline biosynthesis (22). Expression of *thrE* (threonine/serine exporter) in the blue module further suggests that *Xp* actively prevents toxic intracellular accumulation of amino acids while fine-tuning osmotic balance. Notably, unlike *Pseudomonas syringae*, which primarily upregulates amino acid transporters *in planta* (22), *Xp* engages both uptake and biosynthetic routes early in apoplastic colonization. This may suggest the pathogen’s strategy (*Xp*) to prioritize metabolic autonomy, securing key amino acid precursors, maintaining redox-osmotic homeostasis, while also equipping itself with the translational capacity to fulfill the energy demands of virulence.

Genes involved in broader amino acid biosynthesis were distributed across apoplast-associated modules. The yellow module showed strong expression of the *metLB* operon and *metXS*, indicating activation of both canonical and alternative methionine biosynthesis pathways, similar to *Pseudomonas*. Branched-chain amino acid biosynthesis genes (*ilvM, ilvG, ilvC)* and shikimate pathway enzymes (chorismate mutase) were induced across apoplastic infection, while *trpC/trpD* for tryptophan biosynthesis, threonine (*thrABC*) along with *thrE* (a threonine/serine exporter for excess amino acids removal) and arginine (*argHCABEGF*) biosynthesis genes were primarily induced during early apoplast colonization. Early apoplastic colonization also supported proline catabolism, as indicated by expression of *putA*, encoding proline dehydrogenase, where it oxidizes proline to glutamate, providing carbon and nitrogen and linking nitrogen metabolism, osmoprotection, and T3SS induction (103). Histidine biosynthetic genes (*hisA, hisB, hisC, hisD,* and *hisH*) were also induced, consistent with histidine limitation in the apoplast, and the importance of histidine homeostasis for virulence, redox balance, and metal stress tolerance.

#### Carbohydrate acquisition fuels growth and extracellular polysaccharide production

Early apoplastic colonization by *Xp* is characterized by strong induction of carbohydrate acquisition and exopolysaccharide (EPS) biosynthesis. The midnight blue module included *oprB* (formerly *rpfN*)*,* a carbohydrate-selective porin essential for growth and virulence (log_2_FC: 1.50). Loss of this porin reduces sugar uptake and induces compensatory CWDEs to liberate plant-derived sugars, underscoring its role in nutrient acquisition (104). Carbohydrates imported via *oprB* likely fuel EPS production, supported by co-expression of *gumEFGHIJ* in the early apoplast (log_2_FC: 1.20–2.47 relative to 72 h) and *gumKLM* in the yellow module (log_2_FC: ~2.37, relative to epiphyte), suggesting that EPS production begins early and persists throughout colonization, contributing to biofilm formation and protection against host defenses. Additional sugar metabolism genes induced early at 24 h included *scrK* (fructokinase), *fucP* (fucose permease), and sucrose utilization genes within the *sux* operon (*suxA*, *suxC*, and regulator *suxR*), consistent with the importance of sucrose uptake for virulence (105). Induction of succinyl-CoA synthetase (*sucC* and *sucD*), further links carbohydrate assimilation to TCA cycle activity and energy generation during apoplastic infection.

#### Additional metabolic adaptations reflect increasing environmental stress

Early apoplastic colonization was marked by induction of cobalamin (vitamin B_12_) biosynthesis genes (*cobU, cobQ*; log_2_FC: 2.57 relative to 72h), supporting cofactor-dependent central metabolism and redox balance under nutrient limitation. Concurrent upregulation of the F_0_F_1_ ATP synthase (*atpAGDC*)*,* and fatty acid biosynthesis genes (*fabFGDH*), indicates elevated energy investment and membrane remodeling including xanthomonadin pigment production, to maintain envelope integrity under host-induced oxidative stress during early apoplastic colonization. Early infection was further characterized by induction of ribosomal proteins, ribosome maturation factor (*rimP*), RNA polymerase subunits (*rpoBC*), and translation-associated factors, consistent with increased biosynthetic and virulence demands. In contrast, late apoplastic infection induced the *moaEDCAB* operon, which is involved in molybdenum cofactor (Moco) biosynthesis. Enhanced Moco production likely activates molybdoenzymes required for redox regulation, anaerobic respiration, and nitrogen metabolism, facilitating metabolic flexibility and survival as the apoplast becomes increasingly oxygen and nutrient-limited (106). Genes involved in peptidoglycan biosynthesis were strongly represented in the early apoplast-specific blue module, indicating a large investment in robust cell envelope biogenesis. These include the *mur* gene cluster (*murB* through *murF*), which encodes enzymes responsible for sequential assembly of soluble peptidoglycan precursors, culminating in UDP-N-acetylmuramyl-pentapeptide synthesis (107). Co-expression of multiple *fts* family cell division genes further indicates active cell wall remodeling and septation during early apoplastic infection.

#### Virulence systems facilitate host exploitation and nutrient release

Early apoplastic colonization was characterized by strong induction of type II secretion system (T2SS) components and associated CWDEs. These included glycoside hydrolases (GH98, GH2, GH3, GH92, GH95, GH97, GH99), cellulases (*celD*), beta-galactosidases (*galA*, *bga2*), and glycosyltransferases (GT41, GT2), together with core T2SS machinery genes (*xpsFMND*), highlighting active export of plant cell wall degrading enzymes facilitating nutrient acquisition (25, 108). Additional CWDEs uniquely expressed early included GH5, GH28, GH31, GH35 (*galD*), GH43, GH95, GH125, and GH127 (log_2_FC: ~1 to 5 compared with 72h). These CWDEs target diverse substrates, such as cellulose, hemicellulose, and pectin, enabling pathogen to exploit plant derived carbohydrates during infection. Other CWDEs expressed uniquely in apoplast include GH5 cellulase (*egl*), cellulase (*engXCA*), GH3 (*bglX*), and chitinase GH19. Core components of a second T2SS (*xcsH* and *xcsC,* and *xcsF*) were observed throughout the apoplastic infection. Interestingly, some T2SS-related genes and glycosyltransferases (e.g., GT21, GH15, GH30) were expressed during later stages of disease, suggesting a sustained role of T2SS in virulence and tissue maceration as infection progresses.

In parallel with host tissue degradation, genes associated with cell division were co-expressed with T3SS genes and effector repertoires during early apoplastic colonization, indicating coordinated investment in growth and virulence. T3SS genes showed strong induction in early apoplast (avg. log_2_FC ~4 relative to late apoplast and ~5 relative to epiphytic growth). Most core *hrp* cluster genes, including chaperone *hpaB*, together with effectors (*xopV, xopN, xopZ, xopX, xopF1, xopK,* and *avrXv4*), clustered in the blue module, whereas additional effectors (*avrBs2, xopL, xopAD, xopI, xopAE, xopF2*, and *xopAV*) were enriched in the early apoplast-specific midnight blue module. Most core *hrp* cluster genes, including the chaperone *hpaB*, are significantly downregulated under epiphytic conditions compared with the apoplast at 24 h (average log2FC: −5.42), as are effectors *(xopV, xopN, xopZ, xopX, xopF1, xopK, xopAV, xopL, xopAD, xopAE, xopF2,* and *avrXv4)*, with log2FC ranging from −0.92 to −5.02. Notably, several effectors were associated with the purple module expressed at 24 h, while others (*xopAV, avrXv3)* were associated with the apoplast-specific green yellow module, indicating spatiotemporal regulation of effector deployment across infection stages (25, 109). The observation that some effectors were transcribed in the absence of *hrp* gene expression raises the possibility that certain effectors, particularly plasmid-borne ones, may be pre-synthesized and poised for rapid delivery once a functional T3SS is assembled, or may utilize alternative delivery routes, as reported for AvrBs3 homologs (110).

#### The HrpG regulon coordinates virulence and metabolic adaptation

The HrpG core regulon encompassing T3SS structural genes, effectors, and metabolic regulators (*pcaQ, virK,* and *vanK*) was expressed primarily in the early apoplast-associated blue module. Plant cell-wall degradation releases phenolic compounds, including vanillic acid, 4-hydroxybenzoic acid, and hydroxycinnamic acid. VanK mediates their uptake, while PcaQ regulates their catabolism, enabling detoxification and use of aromatic intermediates as carbon sources (111). Additional HrpG core regulon members, including *phoC* (acid phosphatase), and PAP2 superfamily, genes were enriched in the apoplast-associated green yellow module, whereas chorismate mutase was associated with the yellow module, highlighting layered niche-specific deployment of HrpG-regulated functions during apoplastic colonization.

#### Cell-envelope remodeling supports persistence and immune evasion

A strong induction of LPS biosynthesis genes was upregulated during early apoplastic colonization. These genes are located in a conserved cluster between *metB* and *etfA* (E2P69_RS05020-RS05085), encoding enzymes required for assembly of the LPS core and O-antigen (112). In addition, the *rmlABCD* operon, responsible for synthesis of dTDP-L-rhamnose, the direct precursor for rhamnose moieties incorporated into the LPS core and O-antigen, was highly expressed. During late apoplastic infection, induction of LPS export genes (*lptG*, encoding ABC transporter permease) was observed. Together, LPS biosynthesis and export likely support outer-membrane integrity and immune evasion throughout apoplastic colonization, particularly during prolonged colonization under stress conditions (113).

#### Host defenses select for oxidative, osmotic, and envelope stress adaptations

Early apoplastic colonization by *Xp* is marked by activation of detoxification, redox balancing, and stress adaptation pathways that enable survival in the chemically hostile apoplast. The midnight blue module included *ligA* and *ligB*, encoding dual role extradiol catechol dioxygenases that catalyze oxidative cleavage of substituted catechols (67), detoxifying antimicrobial phytoalexins, while generating aromatic intermediates for metabolism (114). Early apoplast samples also showed strong induction of cytochrome c biogenesis genes (*ccmE, ccmG,* and *ccmF*), supporting their critical role in electron transport and oxidative stress tolerance. In parallel, genes from the *dsbE* family, responsible for introducing disulfide bonds into periplasmic proteins, were also co-expressed, ensuring proper folding of virulence factors and stress-response proteins under oxidative conditions. Both ccm and dsb pathways have been implicated in tolerance to reactive oxygen species (ROS) and phenazines (115).

The stringent response was also activated early, as indicated by induction of *spoT*, encoding bifunctional (p)ppGpp synthetase/guanosine-3′,5′-bis(diphosphate) 3′-pyrophosphohydrolase. SpoT controls cellular levels of alarmone nucleotides (p)ppGpp and maintains the balance between synthesis and degradation of (p)ppGpp that is important in adaptation to the changing environment. This global regulatory pathway reprograms transcription under carbon and nitrogen limitation, reducing ribosomal RNA synthesis and prioritizing stress survival and virulence (116, 117). Defense against plant-secreted antimicrobials was evident through strong expression of NADH-quinone oxidoreductase genes (*nuoBCDEFGHIJLMN*) in the blue module, and *nuoA* in the midnight blue module, which mitigates toxicity from plant-derived quinones, while sustaining respiratory metabolism (118, 119). Additional stress-related genes were observed, encoding BatD oxygen tolerance proteins, SmeC multidrug efflux protein, and YicC/YloC family endoribonuclease. The presence of glyoxalase/bleomycin resistance protein/dioxygenase superfamily genes expressed during early apoplast colonization suggests that mechanisms for detoxifying redox-cycling compounds are in place to maintain redox balance (120).

#### Persistent apoplastic colonization requires maintenance of redox balance, osmoprotection, and Fe-S cluster integrity

Pyrroloquinoline quinone (PQQ) biosynthesis genes (*pqqE* and its chaperone *pqqD*) were observed in the apoplast-specific modules. The induction of these genes suggests that *Xp* leverages PQQ-mediated electron transfer to stabilize redox homeostasis under stress conditions (121). The glycine betaine/L-proline transporter *proP* was expressed in the yellow module, specific to the apoplastic niche, and showed significant induction at 24 h compared with 8 h during epiphytic colonization (log_2_FC: 2.72), indicating sustained responses to water limitation. The Suf pathway (*sufD*, *sufC*, and early induction of *sufE*) was activated to maintain iron homeostasis and repair oxidatively damaged Fe-S clusters.

#### Late infection favors survival under chronic oxidative, osmotic, and oxygen-limiting conditions

During late apoplastic infection, additional systems were activated to sustain viability under low-oxygen and nutrient-limited conditions. These included cytochrome bd complex *(cioA,* and *cioB*, log_2_FCs: 3.10 and 3.14 respectively, relative to 24 h apoplast) that enhances resistance to toxic compounds such as metals, nitric oxide, sulfide, cyanide, hydrogen peroxide, and peroxynitrite, supporting survival under oxidative stress and low-oxygen conditions typical of the apoplast. The gene *tolB*, upregulated during late infection, plays a role in TonB-independent uptake and cell envelope stability. Genes encoding the ClpXP protease system and stringent starvation protein (*sspB*) were expressed during late infection, suggesting involvement in protein quality control and stress adaptation. Additionally, *glpR*, a glycerol metabolic repressor that responds to glycerol 3-phosphate (G3P), a crucial module signal of systemic acquired resistance, was expressed during late infection. G3P-mediated relieving of the GlpR-dependent repression of the *glp* gene cluster and imposing of bistable growth pattern (122) suggests that *Xp* may sense and respond to plant-derived G3P, adjusting its metabolic state to optimize survival under stress. A CHASE-domain histidine kinase (*pcrK*) was observed, which senses plant cytokinins and initiates signaling cascades that influence bacterial gene expression and help *Xanthomonas* adapt to the plant environment by altering virulence and improving resistance to oxidative stress (123). Genes encoding polyhydroxyalkanoate (PHA) granule-associated proteins were induced, suggesting that when carbon sources are abundant, but nitrogen is limited, the pathogen stores excess carbon as PHAs. These granules may stabilize membranes under hypertonic conditions, aiding survival during plasmolysis. Additionally, osmoprotectant systems were activated, including, *yehX/yehZ*, glycine betaine transporters for osmotic stress tolerance, *egtB* involved in ergothioneine biosynthesis, and alpha-trehalose phosphate synthase, supporting trehalose-mediated osmoprotection. Genes encoding cupin-domain protein, phosphomannose isomerase, were expressed alongside sterol-binding proteins and regulators of ubiquinone biosynthesis (protein kinase regulator of UbiI) that contribute to LPS assembly and membrane integrity, essential for virulence and phage resistance (124, 125). The apoplast undergoes an oxidative burst during infection, driven by plant defenses. The oxidation of apoplastic ascorbic acid by ascorbate oxidase alerts redox signaling, and its conversion to oxalic acid may further restrict pathogen growth. Genes encoding *sodC1* (superoxide dismutase) and catalases *(katE*, Mn-catalase) are expressed to neutralize ROS and withstand oxidative stress (126). Interestingly, these oxidative stress response genes differ from those expressed in the epiphytic niche, indicating niche-specific compartmentalization of stress adaptation strategies in *Xp*.

### Stress-responsive shared flagellar motility and chemotactic navigation gene expression between late apoplastic and epiphytic phases indicate a transitional stage for pathogen dissemination and surface recolonization

The magenta module was associated with late apoplast infection and both epiphytic time points. Of the 341 genes, 328 were significantly upregulated during late apoplast infection relative to early infection, whereas only 69 showed significant differential expression between epiphytic time points, indicating largely similar expression profiles across epiphytic conditions (see Table S4 for log_2_FC values). This module contained a complete apparatus of flagellar motility genes, including all the structural components (E2P69_RS17025 through RS17080; RS17170 to RS17260) being majorly upregulated during apoplast 72h (log_2_FC: 3-4.5 relative to 24h), whereas, motor proteins are upregulated during early epiphyte and late apoplast conditions only, driving the flagellar rotation. Further, chemotaxis and multiple chemosensory components (*cheY, cheR2, cheD, cheB, cheY, cheZ, cheA, cheW, cheA2*) suggest a coordinated reactivation of swimming motility and chemotactic navigation during late apoplastic and early epiphytic infection. This pattern mirrors the hierarchical architecture of flagellar biogenesis described in *Xanthomonas campestris*, where RpoN1 (σ⁵⁴) and FleQ initiate expression of FT3SS, rod, and hook genes, followed by FliA (σ²⁸) for filament assembly, with FlhA/FlhB/FlgM gating export and sigma-factor activity. Perturbation of FlhA/FlhB reduces infection, underscoring the virulence link (127). Co-expression of *cheY* and additional sensory systems includes three PAS-domain proteins (RS02915, RS11650, RS12280), two HAMP-domain proteins (RS17605, RS19020), and two STAS-domain proteins (RS20270, RS22520), which integrate environmental cues into motility regulation, support re-establishment of directional sensing, and chemotactic bias during late apoplast navigation (128). The chemotaxis protein in this module and in the green module also showed a decline by the late epiphytic phase (24 h). Additionally, the magenta module also encoded multiple c-di-GMP signaling components, including four EAL-domain proteins (E2P69_RS09020, RS17280, RS17845, E2P69_RS20925), five GGDEF-domain diguanylate cyclases (RS10540, RS10550, RS18400, RS22020, & RS22030), and a single PilZ-domain effector (RS17160) located near flagellar genes, indicating fine-tuning of motility and biofilm dispersal in response to intracellular c-di-GMP levels. The lack of YcgR/cellulose biosynthesis brakes in the module (which shows upregulation at 8h after dip-inoculation in the green module), combined with increased expression of flagellar and chemotaxis genes (upregulation in the magenta module), allows the pathogen to transition to the egression phase through stomata (4).

Additional sensory systems include three PAS-domain proteins (RS02915, RS11650, RS12280), two HAMP-domain proteins (RS17605, RS19020), and two STAS-domain proteins (RS20270, RS22520), which integrate environmental cues into motility regulation. Another oxygen and stress sensing histidine kinase induced in this phase was a PAS-domain hybrid histidine kinase homologous to StoS (XOO_0635 in *X. oryzae*), an O₂ sensor, whose deletion reduces tolerance to high osmolarity, sodium, and H₂O₂ and diminishes *in planta* fitness (129). Other stress adaptation genes expressed in this module include glutathione-S-transferase, glutaredoxin, and a glutathione-dependent formaldehyde-activating enzyme, which collectively support redox homeostasis. Antioxidant defenses are further reinforced by the expression of *srpA* (catalase family peroxidase) and genes encoding carboxymuconolactone decarboxylase family proteins, which are antioxidant proteins with alkyl hydroperoxidase activity. Components required for detoxifying reactive oxygen species include AhpC-associated systems, which depend on thiol reduction for regeneration, SodM (superoxide dismutase), and thioredoxin (TrxA), ensuring protection against oxidative damage and maintenance of protein integrity.

This shared pattern of gene expression is consistent with the conceptual model proposed by Beattie and Lindow (4), in which bacterial populations exit the leaf interior through stomata during later stages of infection and re-establish themselves on the leaf surface. In this context, the late apoplastic phase represents not only continued colonization within plant tissues but also preparation for a return to an epiphytic lifestyle as a part of egression. Supporting this interpretation, the magenta module contained numerous flagellar motility genes together with genes involved in stress tolerance and environmental adaptation. The coordinated expression of these functions indicates bacterial relocation to the phyllosphere, where motility facilitates surface dispersal and stress-response mechanisms promote survival under fluctuating environmental conditions. Collectively, transcriptional programs active during late apoplastic colonization partially overlap with those required for epiphytic persistence, reflecting the transition from endophytic growth back to a surface-associated lifestyle (39).

### Conclusion

**Fig 4:**
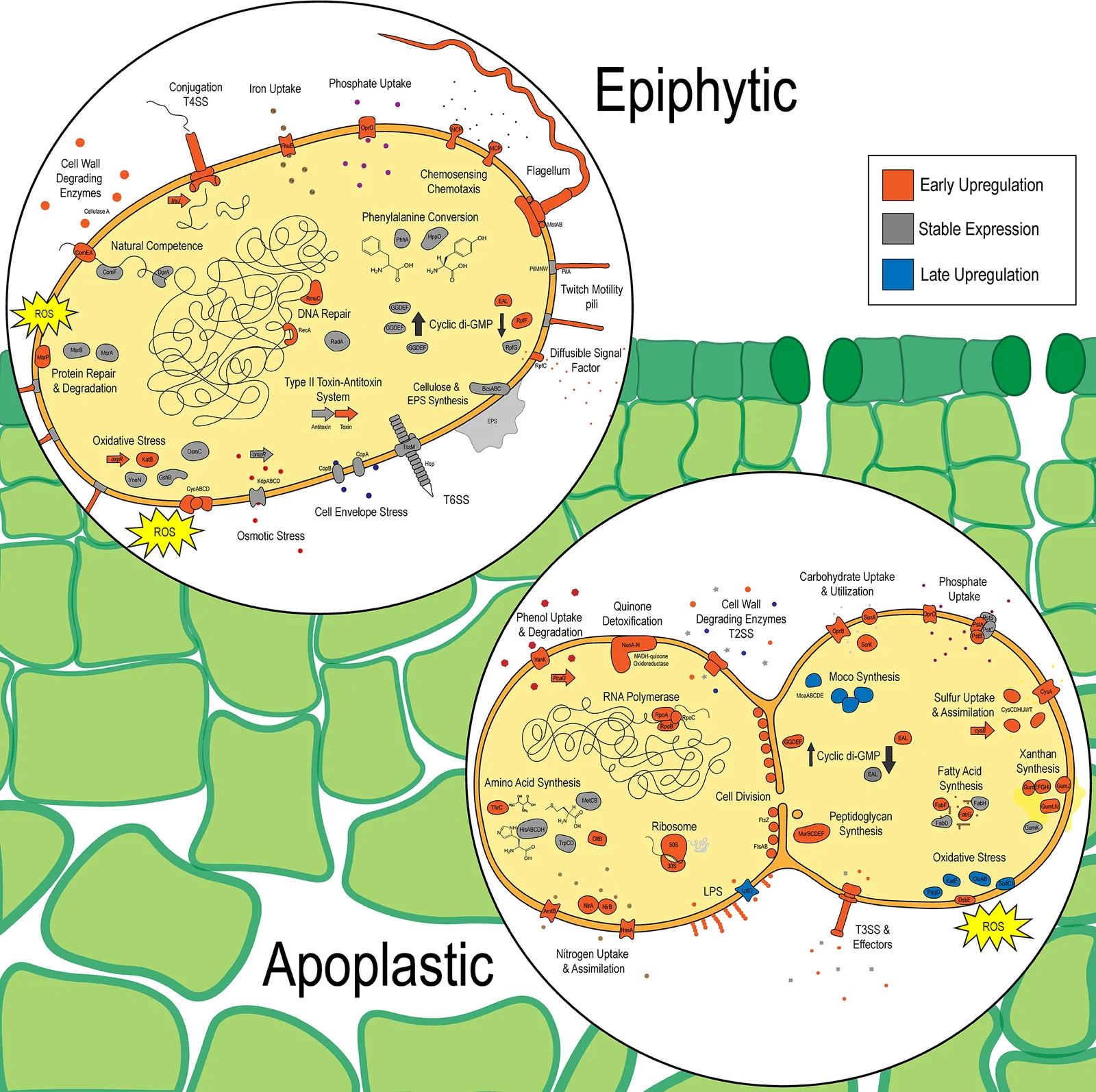
Summary of cellular systems upregulated only in epiphytic or apoplast conditions. Not all upregulated systems and genes are shown. Components experiencing significant upregulation according to DESeq in the early time point (8 h for epiphytic and 24 h for apoplast) are colored in orange, significant upregulation in the late time point (24 h for epiphytic and 72 h for apoplast) are colored in blue, and components that were not significantly different between time points are shown in gray.

This study revealed remarkably distinct patterns of RNA transcript expression both spatially and temporally for *Xanthomonas perforans* infection on tomato. This not only provides clues about the conditions that the pathogen encounters both epiphytically and apoplastically but also demonstrates the importance of these dual lifestyles and the ability to rapidly transition between lifestyles to successfully colonize the leaf, cause disease, and be able to disseminate. Here, we present a coherent model that summarizes the niche-specific and infection-stage-specific gene expression patterns (Fig. 4). The early epiphytic lifestyle is characterized by chemosensing, motility, including both flagellar and twitching motility, uptake of scarce nutrients such as iron and phosphate, production of DSF and cellulose, conjugation, natural competence, and DNA repair. The overall epiphytic lifestyle also includes upregulation of genes to counter oxidative stress and repair or degrade damaged proteins, reduce osmotic stress through potassium pumps, synthesize cellulose, type II toxin-antitoxin systems, copper resistance proteins, the type VI secretion system, and genes suggesting a greater focus on c-di-GMP synthesis compared with degradation. Alternatively, in the early apoplast the pathogen upregulates genes for reproduction or cell turnover, including nitrogen uptake and assimilation, amino acid synthesis, RNA polymerase, ribosomes, carbohydrate uptake, and proteins for cell division organization and peptidoglycan synthesis while favoring genes for degradation of c-di-GMP compared with synthesis. Additionally, the early apoplastic lifestyle is characterized by virulence traits, T2SS, CWDE, and T3SS and effectors while also showing signs of defense from the plant’s immune response, including increased phenol uptake and degradation, quinone detoxification, the synthesis of fatty acids, xanthan, and LPS, in addition to the upregulated uptake of phosphate and sulfur to counter the plant’s sequestration of nutrients in response to infection. The late apoplast is defined by upregulated genes for molybdenum cofactor synthesis, for ROS detoxification such as catalase, and increased transmembrane transport of LPS. Further, WGCNA analysis revealed unique patterns of gene expression with modules specific to early colonization regardless of location, revealing co-expression of genes for type four pili, conjugation, and plasmid-borne effectors. Another module showed co-expression of flagella and chemotaxis genes for epiphytic and late apoplastic potentially showing these functions are of particular importance for the transition phase from the apoplast to the epiphytic surface for dissemination. Overall, the majority of epiphytic and apoplastic traits in *Xp* important for tomato colonization closely mirrored those observed in *P. syringae* on bean leaves. Notable differences, however, were evident in niche-specific metabolic and stress response strategies. In *Xp*, sulfur metabolism-related genes were co-expressed upon entry into the apoplastic niche, in contrast to *P. syringae*, where they were predominantly upregulated in the epiphytic niche. Additionally, oxidative stress-related genes in *P. syringae* were induced mainly after transition into the apoplast, whereas, *Xp* exhibited distinct and non-overlapping sets of oxidative stress-related genes between the epiphytic and apoplastic niches. While this study reveals information into the different traits expressed during the lifecycle of this devastating bacterial spot pathogen, there is still more knowledge yet to be gained about the triggers and pathways for the initialization of the transition between these life stages to elucidate the full picture of this pathogen’s ability to infect its host.

## MATERIALS AND METHODS

### Plant material, bacterial strain, and growth conditions

The tomato (*Solanum lycopersicum*) cultivar FL-47 was used in this study. Two-week-old seedlings were transplanted into 4-inch pots with soil-less standard premix potting medium. Plants were grown at 28 to 30°C under greenhouse conditions. For the experiment, 4- to 5-week-old plants were used. The *Xanthomonas perforans* (*Xp*) strain used in this experiment was AL65 (GCF_007714115.1), originally isolated from pepper (*Capsicum annuum*) in Alabama, USA (130). The strain was routinely grown in nutrient medium at 28°C with or without agitation.

### *In planta* bacterial growth for RNA extraction

The AL65 strain was streaked on nutrient agar plates and grown for 36 h at 28°C. The cells were scraped off the plates using sterile 0.01M MgSO_4_ for inoculum preparation. The cells were washed two times using sterile 0.01M MgSO_4_. The workflow for *in planta* transcriptome is shown in Fig. 1. To establish an epiphytic population, the inoculum density was adjusted to ~ 10^8^ CFU/mL (OD_600nm_ = 0.3) and amended with 0.01M MgSO_4_ containing 0.025% Silwet, and the plants were dipped into the inoculum for 30 seconds such that the abaxial and adaxial surfaces of the leaves were completely immersed in the inoculum. The dip-inoculated plants were maintained in greenhouse conditions, and leaves were collected at 8 and 24 h post-infection. The inoculated plants were kept in the custom-built humidity chambers in the greenhouse. Each humidity chamber frame was built with PVC pipes and covered with transparent polyethylene sheets from all sides. Inside each chamber, high relative humidity was maintained by using an overhead sprinkler irrigation system with irrigation set to water at 8am and 6pm for 1 minute. These conditions sustained ~80% relative humidity (Fig. 1). Relative humidity and temperature were monitored using a HOBO Data logger during the experiment. A total of 500–600 primary leaves were collected, immediately submerged in 2-L RNA stabilization solution (10 mL water-saturated phenol [pH < 7.0], 190 mL ethanol, 1.8 L water), sonicated for 10 min, and centrifuged at 5,000 × *g* for 10 min to collect bacterial cells. The cell pellets were suspended in residual supernatant and filtered through a 5-μm filter (Millex-SV syringe filter unit; Millipore Corp.). The cells in the filtrate were harvested by centrifugation at 5,000 × *g* for 5 min, the supernatant was discarded, and the pellets were placed at −20°C. The cells collected from the 500–600 leaflets from 50 plants per replicate at a time point served as a biological replicate, and the procedure was repeated to provide three biological replicates for each time point.

To establish apoplastic populations, bacteria were introduced into the plant by vacuum infiltrating leaves submerged in the inoculum. The plants and bacteria were grown, and the inoculum was prepared, as described above. We used a different inoculum density for each time point, 10^7^ CFU/mL for 24 h time point to allow recovery of enough cells during the early time point of 24 h post-inoculation and 10^5^ CFU/mL for the 72 h time point to capture pathogen gene expression during later stages of disease development while avoiding necrosis of the plants before the 72 h sampling point. Infiltrated plants were incubated for 24 and 72 h in greenhouse conditions. A total of 80–100 leaves were collected and submerged immediately in an acidic phenol RNA-stabilizing solution as described above. The leaves were cut into squares (∼3 × 3 mm^2^) while submerged, and the plant tissues and liquid were sonicated for 10 min. The solution was filtered through a 70-μm filter with repeated filter changes to remove host contamination or debris. The filtrate was centrifuged at 7,000 × *g* for 10 min, and the pellet was suspended in the remaining supernatant, which was subjected further to three washings with sterile ddH_2_O to eliminate plant material. The suspension was filtered through a 5-μm filter with repeated filter changes. The bacterial cells were harvested by centrifugation at 7,000 × *g* for 10 min, the supernatant was discarded, and the pellets were flash frozen and placed at −20°C. The cells collected from the 80–100 leaves at a single time point served as a biological replicate, and three biological replicates were generated at each time point.

### RNA extraction, ribodepletion, and sequencing

Total RNA was extracted using TRIzol (Thermo Fisher Scientific, Waltham, MA, USA) as described previously (131). The concentration of RNA samples was determined using Qubit RNA HS Assay kit (Invitrogen). The ribodepletion was carried out at the sequencing center (SeqCenter), targeting the plant host RNA, primarily abundant rRNA in our sample, through hybridization to customized DNA probes, which are listed in Table S1. Sequencing was done on a NovaSeq X Plus, producing paired-end 150 bp reads. Demultiplexing, quality control, and adapter trimming were performed with bcl-convert (v4.2.4).

### Analysis of RNA-Seq results

The sequenced data were processed to remove adapters, and the FASTQ files were generated. We used the epinatel pipeline (https://github.com/epinatel/Bacterial_RNAseq/). The pipeline uses steps involving read quality filtering using Trimmomatic (132), mapping to the reference genome of *Xp* AL65 (RefSeq: NZ_SMVI00000000.1, assembly: GCF_007714115.1) using Bowtie2 (133). Differentially expressed genes (DEGs) across the four treatment conditions (apoplastic and epiphytic each with two time points) were identified using the DESeq2 R package (v1.46.0) (134). This method analyzes RNA-seq count data using a model based on the negative binomial distribution to detect differences in gene expression between treatments. The resulting *p*-values were adjusted for multiple testing using the Benjamini–Hochberg method to control the false discovery rate (FDR). Genes with an adjusted *P*-value of less than 0.05 were considered significantly differentially expressed and were used for further functional enrichment analysis. Gene ontology analysis was performed using EggNOG (v.5.0) (135). KEGG pathway enrichment analysis was performed on the differentially expressed genes using the clusterProfiler R package (v4.14.6). This analysis was used to identify biological pathways that were significantly overrepresented among the differentially expressed genes. KEGG pathways with an adjusted *P*-value of less than 0.05 were considered significantly enriched (http://www.genome.jp/kegg/).

### Gene co-expression network analysis

Gene co-expression network analysis was performed using RStudio with the WGCNA package (v1.73) (136). Gene read counts were filtered to retain genes with ≥10 reads in at least three samples and then normalized using the variance stabilizing transformation from the DESeq2 package (v1.49.4). The normalization accounted for differences in sequencing depth across apoplast vs epiphytic conditions. To construct the co-expression network, a soft-threshold power (β = 7) was chosen, which was the first value to reach a correlation coefficient of 0.82, for computing the adjacency matrix (measuring connection strength between genes). This matrix was then used to calculate the topological overlap measure (TOM), using the “signed” option to ensure modules capture biologically meaningful patterns where genes exhibit coordinated up- or down-regulation. Pairwise gene distances derived from TOM were used to perform hierarchical clustering, and modules were initially identified using the cutreeDynamic function with deepSplit = 2 and a minimum module size of 30. Initially, 17 modules were observed, which were subsequently merged based on ≥ 75% similarity (MEDissThres = 0.25) using the mergeCloseModules function, resulting in a final set of 8 co-expression modules. Module–trait relationships were assessed using Pearson’s correlation. Top genes within each module were selected based on a module membership (kME) value greater than 0.8, and correlations with *P*-value < 0.05 were considered statistically significant.

## Data Availability

All samples are available in the NCBI Sequence Read Archive under the BioProject accession number PRJNA1405621. All codes and output files from different analyses conducted are uploaded on GitHub (https://github.com/Potnislab/Pathogen_inplanta-transcriptome.git).

## SUPPLEMENTAL MATERIAL

Supplemental material aem.00681-26-s0001.xlsx Tables S1 to S4.

ASM does not own the copyrights to Supplemental Material that may be linked to, or accessed through, an article. The authors have granted ASM a non-exclusive, world-wide license to publish the Supplemental Material files. Please contact the corresponding author directly for reuse.
